# Exploring Regorafenib Responsiveness and Uncovering Molecular Mechanisms in Recurrent Glioblastoma Tumors through Longitudinal In Vitro Sampling

**DOI:** 10.3390/cells13060487

**Published:** 2024-03-11

**Authors:** Mariangela Morelli, Francesca Lessi, Sara Franceschi, Gianmarco Ferri, Manuel Giacomarra, Michele Menicagli, Carlo Gambacciani, Francesco Pieri, Francesco Pasqualetti, Nicola Montemurro, Paolo Aretini, Orazio Santo Santonocito, Anna Luisa Di Stefano, Chiara Maria Mazzanti

**Affiliations:** 1 Fondazione Pisana per la Scienza, San Giuliano Terme, 56017 Pisa, Italy; f.lessi@fpscience.it (F.L.); s.franceschi@fpscience.it (S.F.); m.giacomarra@fpscience.it (M.G.); c.mazzanti@fpscience.it (C.M.M.); 2 Neurosurgical Department of Spedali Riuniti di Livorno, 57124 Livorno, Italyorazio.santonocito@uslnordovest.toscana.it (O.S.S.);; 3 Radiotherapy Department, Azienda Ospedaliera Universitaria Pisana, 56126 Pisa, Italy; 4 Department of Neurosurgery, Azienda Ospedaliera Universitaria Pisana, 56126 Pisa, Italy; nicola.montemurro@unipi.it

**Keywords:** glioblastoma, NADP(H) FLIM, Regorafenib, drug response, organoids

## Abstract

Glioblastoma, a deadly brain tumor, shows limited response to standard therapies like temozolomide (TMZ). Recent findings from the REGOMA trial underscore a significant survival improvement offered by Regorafenib (REGO) in recurrent glioblastoma. Our study aimed to propose a 3D ex vivo drug response precision medicine approach to investigate recurrent glioblastoma sensitivity to REGO and elucidate the underlying molecular mechanisms involved in tumor resistance or responsiveness to treatment. Three-dimensional glioblastoma organoids (GB-EXPs) obtained from 18 patients’ resected recurrent glioblastoma tumors were treated with TMZ and REGO. Drug responses were evaluated using NAD(P)H FLIM, stratifying tumors as responders (Resp) or non-responders (NRs). Whole-exome sequencing was performed on 16 tissue samples, and whole-transcriptome analysis on 13 GB-EXPs treated and untreated. We found 35% (n = 9) and 77% (n = 20) of tumors responded to TMZ and REGO, respectively, with no instances of TMZ-Resp being REGO-NRs. Exome analysis revealed a unique mutational profile in REGO-Resp tumors compared to NR tumors. Transcriptome analysis identified distinct expression patterns in Resp and NR tumors, impacting Rho GTPase and NOTCH signaling, known to be involved in drug response. In conclusion, recurrent glioblastoma tumors were more responsive to REGO compared to TMZ treatment. Importantly, our approach enables a comprehensive longitudinal exploration of the molecular changes induced by treatment, unveiling promising biomarkers indicative of drug response.

## 1. Introduction

Glioblastoma (GB) stands out as the most prevalent malignant primary brain tumor affecting adults [1]. The standard treatment protocol includes maximal surgery followed by temozolomide (TMZ) treatment and radiotherapy. However, the median survival is currently 14.6 months [2], with GB relapsing in nearly all patients around 6–9 months after the initial therapy [3,4]. Currently, managing disease recurrence poses a significant challenge.

Multikinase inhibitors, compounds designed to target a variety of kinases, have been examined in numerous studies for the treatment of recurrent tumors [5,6]. The objective was to address diverse tumor-related pathways, encompassing invasion and metastasis, cell growth and survival, and neo-angiogenesis. Regorafenib (REGO), among the multikinase inhibitors, is presently utilized in clinical settings for the treatment of hepatocellular carcinoma, gastrointestinal stromal tumors, and colorectal cancer [7,8,9]. Recently, in a phase II clinical trial focused on recurrent GB (REGOMA), this drug demonstrated promising and noteworthy results [10]. The study involved 119 patients with recurrent GB, revealing extended overall survival (OS) (7.4 months compared to 5.6 months with lomustine). Notably, the REGO arm exhibited a statistically significant 6-month improvement in progression-free survival (PFS) compared to the lomustine-treated group, as reported in the clinical trial. Based on these findings, the National Comprehensive Cancer Network (NCCN) 2020 Guidelines designated REGO as a preferred regimen for treating relapsed GB, and the Italian Agency of Medicine (AIFA) granted approval for its use in Italian patients with recurrent GB [11]. From a molecular perspective, the angiogenic VEGFR 1–3, PDGFR-b, and the oncogenic c-KIT, RET, FGFR, and Raf kinases represent REGO targets [12]. However, despite REGO becoming a part of clinical practice as a treatment option for relapsed GB, our comprehension of the molecular mechanisms governing GBM patients’ sensitivity to REGO remains restricted [11]. The limited comprehension of the biological factors influencing a specific drug’s efficacy often results in trials that initially hold promise during early development but subsequently prove ineffective in later stages. Gaining insight into the biological underpinnings of these setbacks can be achieved by collecting tissue samples both before and after treatment. However, the routine practice of gathering such samples has yet to be established in the realms of neurosurgery and neuro-oncology drug development [13]. In pursuit of addressing this pressing need, we have recently pioneered an ex vivo drug response functional precision medicine approach. This innovative methodology allows us to assess how tumor samples respond to various cancer treatments, enabling us to analyze tissue samples both prior to and after treatment [14]. This approach involves the use of an in vitro 3D organoid model derived from vital patient glioblastoma tissue (referred to as GB-EXPs). These organoids are minimally manipulated and cultured briefly to maintain the tumor microenvironment. We leverage the fluorescence properties of NAD(P)H, a cellular enzyme cofactor, using fluorescence lifetime imaging (FLIM). NAD(P)H may be present either bound to proteins or in a free state within cells, and these conditions impact its fluorescence lifetime decay. As previously shown [14], the shift in lifetime distribution towards lower free-/bound-NADP(H) fractions is indicative of a responsive phenotype [14]. Our research primarily centers on GB-EXPs derived from patients’ recurrent tumors. We employ this model to investigate changes in the transcriptome of GB-EXPs after treatment through longitudinal in vitro tissue sampling. Additionally, we establish correlations between transcriptome or exome profile and response to REGO. Importantly, this study represents the first exploration of alterations in gene expression resulting from REGO drug treatments in patient-derived GB organoids. Our findings hold significant promise for advancing personalized precision medicine in the field.

## 2. Materials and Methods

### 2.1. GB Tissue Collection

The research was conducted in compliance with the principles outlined in the Declaration of Helsinki, and the protocol for sample collection received approval from the Ethics Committee of the University Hospital of Pisa (787/2015). Tumor specimens were sourced from 18 patients who had undergone surgical resection of histologically confirmed GBM after providing informed consent. Samples were acquired from either the Neurosurgery Department of the “Azienda Ospedaliero-Universitaria Pisana” or the Unit of Neurosurgery of Livorno Civil Hospital. All patients had a GB diagnosis without a prior history of brain neoplasia and did not exhibit R132 IDH1 or R172 IDH2 mutations. In three out of the eighteen patients, neurosurgeons utilized neuronavigation-guided microsurgical techniques to collect both core and peripheral tumor samples. Peripheral tumor samples were obtained at the initial identification of GB during surgery, while core tumor samples were taken from the resected tumor mass. In cases where the tumor displayed a significant area of central necrosis, samples were collected from tumor regions outside the necrotic area. Patient clinical and demographic data are shown in Table 1. Resected tumors were put in MACS tissue storage solution (Miltenyi Biotec, Bergisch Gladbach, Germany) at 4 °C for 2–4 h. Each tumor specimen was rinsed with Dulbecco’s phosphate-buffered saline (DPBS) (Gibco, Grand Island, NE, USA; New York, NY, USA), within a sterile dish and divided into ~0.5–1 mm^2^ pieces under a biological hood. In an effort to minimize variability due to sampling, we pooled 2–4 pieces of parental tumor tissue into one sample for the subsequent analysis.

Biopsies not immediately processed for GB-EXP cultures were cryopreserved in 90% fetal bovine serum (FBS) (Thermo Fisher Scientific, Waltham, MA, USA) and 1% dimethyl sulfoxide (DMSO) (Thermo Fisher Scientific, Waltham, MA, USA) at −140 °C. Tumor samples designated for histological analysis were promptly fixed in 10% formalin and embedded in paraffin, while portions allocated for additional analyses were stored at −80 °C.

### 2.2. GB Explant (GB-EXP) Cultures

The methodology employed in generating explant cultures for this study was previously described [14], but with some modifications. Briefly, fresh GB tumors or frozen samples, following rapid thawing in a 37 °C water bath, underwent washing with DPBS within a sterile dish and were finely sectioned using a scalpel. The resulting minced tissues were then passed through a 300-micron cell strainer to eliminate larger tissue fragments. Suspension was centrifuged at 1200 rpm for 5′. The pellet was resuspended in 3 mL eBiosceince 1X RBC Lysis Buffer (Invitrogen by Thermo Fisher Scientific, Waltham, MA, USA) for 5′ at room temperature. DPBS was then added to inactive red blood lysis and suspension was centrifuged at 1200 rpm for 5′. Pellet was resuspended in 1200 μL of explant medium, composed of 89% DMEM:F12 without red phenol (Thermo Fisher Scientific, Waltham, MA, USA), 10% FBS (Thermo Fisher Scientific, Waltham, MA, USA), and 1% PenStrep (Thermo Fisher Scientific, Waltham, MA, USA). Two volumes of Vitrogel ORG-4 (TheWell Bioscience, North Brunswick, NJ, USA) was added. The solution obtained was put in an 8-well chamber slide’s coverglass (Nalge Nunc International, Rochester, New York, USA), with 150 µL in each well. Vitrogel was permitted to solidify for 20 min at 37 °C, followed by the addition of 300 µL of medium. Subsequently, the cultures were placed within a sterile incubator maintained at 37 °C, 5% CO_2_, and 90% humidity.

### 2.3. GB Cell Lines

The T98G, U118, and U87 GB cell lines were purchased from the American Type Culture Collection (ATCC, Rockville, MD, USA). These cell lines were cultured in Dulbecco’s Modified Eagle Medium (DMEM, Thermo Fisher Scientific, Waltham, MA, USA) devoid of red phenol and supplemented with 10% FBS and 1% penicillin–streptomycin. For FLIM experiments, cells were cultivated in 35 mm Nunc Glass Bottom Dishes (Thermo Fisher Scientific, Waltham, MA, USA).

### 2.4. Spheroid Cultures

Hanging drop method was used to generate spheroids, using T98G, U118, and U87 GB commercial cell lines, as previously described [15].

### 2.5. Drug Treatments

Regorafenib (Tebu-Bio, Milano, Italy) and TMZ (Sigma, St. Louis, MO, USA) were dissolved in DMSO to create stock concentrations of 20 mM and 50 mM, respectively.

Two-dimensional cell lines were subjected to treatment when they reached 30% confluence, while spheroids were treated the following day after being cultured in vitrogel. The medium was replaced with fresh medium containing either 10, 50, or 100 μM of REGO for treated cells, or an equivalent volume of DMSO for control groups.

GB-EXPs were subjected to treatment 3 days after being cultured. The volume of the medium was substituted with fresh medium containing either TMZ at 600 μM, as detailed previously [14], or REGO at 50 μM for treated explants, with an equivalent volume of DMSO for control samples. Both cells and GB-EXPs underwent treatment for a duration of 72 h.

### 2.6. Cell Viability

For the assessment of cell viability in 2D cell lines, the WST-1 assay (Clontech Laboratories, Mountain View, CA, USA) was employed, while the CellTiter-Glo 3D Cell Viability Assay (Promega, Madison, WI, USA) was utilized for spheroids, following the respective manufacturer’s protocols.

In the 2D model, 5000 cells per well were seeded in a 96-well plate format. WST1 was added 72 h following REGO treatment. The quantification of metabolically active cells was performed by assessing the absorbance at 450 nm using a multiwell plate reader (Tecan, Mannedorf, Switzerland). Optical density (OD) values were normalized to those of non-treated cells (controls). For the 3D model, 2 spheroids per well were seeded in an ultralow attachment 96-well plate for luminescence. CellTiter-Glo 3D reagent was added 72 h after REGO treatment. Luminescence was recorded using Tecan multiwall plate reader. Each assay was conducted in triplicate.

### 2.7. Nucleic Acids Isolation

Genomic DNA was extracted from the original tissue sample, which was stored at −80 °C, using Maxwell 16 Tissue LEV DNA Purification Kit (Promega, Madison, WI, USA), following the manufacturer’s instructions. RNA extraction from GB-EXPs and 3D cell lines was performed with Maxwell 16 LEV Simply RNA Tissue Kit (Promega, Madison, WI, USA), following manufacturer’s protocol.

The concentrations of DNA and RNA were assessed utilizing the Qubit Fluorometer (Life Technologies, Carlsbad, CA, USA), and their quality was evaluated using the Agilent 2200 Tapestation (Agilent Technologies, Santa Clara, CA, USA) system.

### 2.8. KI67 Expression Analysis

Complementary DNA (cDNA) was synthesized from 2 ng of total RNA using the iScript cDNA Synthesis Kit (Bio-Rad, Hercules, CA, USA), following the manufacturer’s protocol, in a final volume of 20 μL. For the analysis of Ki67 expression in cell lines, semiquantitative real-time PCR was conducted in a 10 µL reaction mixture containing 5 µL of SsoAdvanced Universal SYBR Green supermix (Bio-Rad, Hercules, CA, USA), 1 µL of primer Assay (Bio-Rad, Hercules, CA, USA), 2.0 µL of cDNA, and 2 µL of nuclease-free water. We used Human Mki67 PrimePCR SYBR Green Assay (Bio-Rad, Hercules, CA, USA) for Ki67 and Human ACTB PrimePCR™ SYBR^®^ Green Assay (Bio-Rad, Hercules, CA, USA) for B-actin housekeeping gene. PCR amplification was conducted using the CFX96 Touch Deep Well PCR system (Bio-Rad, Hercules, CA, USA) with an initial template denaturation at 98 °C for 30 s, followed by 40 cycles of denaturation at 98 °C for 15 s and annealing at 60 °C for 30 s. Every sample was evaluated in triplicate, with positive and negative controls run concurrently in each reaction. Following amplification, melting curve analysis was performed to evaluate PCR product specificity. Data analysis was carried out using the 2^−ΔΔCT^ method for relative quantification [16].

### 2.9. Confocal Imaging

Images were captured using the Olympus Fluoview 3000 confocal microscope, which is furnished with four laser lines (405/488/561/640 nm), 2 hybrid detectors and 2 standard detectors (Olympus, FV31-HSD and FV31-SD, Tokyo, Japan), using a quadriband 405/488/561/640 nm dichroic mirror (Chroma, Lititz, PA, USA) and a UPLXAPO20X (20X magnification, N.A. = 0.80) for brightfield acquisition or UPLXAPO60XO (60X magnification, N:A. = 1.42) oil immersion objective for FLIM. Confocal pinhole diameter was set to 1 Airy.

### 2.10. Lifetime Imaging

Fluorescence lifetime imaging was performed using MultiHarp 150 (Picoquant, Berlin, Germany) time-correlated single-photon counting (TCSPC) unit and a 405 nm LDH-P-C-375B (Picoquant) excitation laser, powered by a PDL 828 “Sepia II” laser driver (Picoquant) and interfaced through fiber port with the confocal setup previously described. Fluorescence was collected with two PMA hybrid detectors (Picoquant) using a dichroic filter (510 nm) and band pass filters 440/40 for NAD(P)H. Laser pulse frequency was set to 40 MHz, pixel dwell time was set to 10 µs, and 240 cycles of acquisition were performed for each field. Image sizes were of 512 × 512 pixels. Temporal resolution was 80 ps. We obtained between 15 and 20 FLIM measurements for both control and treated samples.

FLIM data were analyzed according Phasor approach, using SimFCS suite (Globals for Images, Laboratory for Fluorescence Dynamics, Irvine, CA, USA). In brief, a mono-exponential lifetime standard (fluorescein (Sigma-Aldrich, St. Louis, MO, USA, 46955), 1 µM in NaOH 0.01M, pH12, t = 4 ns) was first acquired in order to reference the other acquisitions and calibrate the universal circle. This was conducted on SimFCS 2. First, the fluorescein phasor was auto-centered, and then, the acquisition files were all referenced. All data analyses of referenced files were performed using SimFCS 4 as previously described [14,17]

### 2.11. Histology and Staining

Tissues were fixed for 24 h in 10% neutral-buffered formalin (Sigma-Aldrich, Saint Louis, MO, USA) at room temperature, processed through a graded-ethanol series followed by xylene, and embedded in paraffin. Explants, whether in vitrogel or in suspension, underwent the same fixation process before embedding in paraffin. Paraffin-embedded sample sections (5 μm) were stained with hematoxylin (Diapath C0303, Martinengo, Italy) for 40 s and with eosin (Diapath C0353, Martinengo, Italy) for 30 s.

For the immunohistochemical staining process, paraffin slides underwent deparaffinization, followed by antigen retrieval achieved through the use of Epitope Retrieval Solution (pH = 8) (Leica Microsystems RE 716 CE, Wetzlar, Germany). Samples were incubated with Ki67 monoclonal (SP6) (MA5-14520, Thermo Fisher Scientific, Waltham, MA, USA) primary antibody using a dilution of 1:50 for 1 h at RT. Detection of bound antibody was accomplished with the Rabbit Specific HRP/DAP Detection IHC Kit (ab64261 Abcam, Cambridge, UK). Immunohistological and H&E pictures were taken with microscope (CARL ZEISS Axio Observer Z1FLMot, Jena, Germany) after mounting with mounting medium (Fisher Scientific, Miami, FL, USA).

### 2.12. Whole-Transcriptome RNA Analysis (WTA) Libraries

NextSeq 500 (Illumina, San Diego, CA, USA) was used for RNA-Seq. The libraries ware prepared using Illumina Stranded Total RNA Prep with Ribo-Zero Plus kit (Illumina, San Diego, CA, USA), starting from 100 ng of total RNA, according to manufacturer’s protocol. The libraries were quantified using Qubit reagents (Thermo Fisher Scientific, Waltham, MA, USA) and analyzed for validation through TapeStation (Agilent, Headquarters, Santa Clara, CA, USA). A maximum of 10 libraries were loaded on NextSeq High Output cartridge (150 cycles).

### 2.13. Whole-Exome Analysis (WEA) Libraries

Whole-exome library preparation was performed using Illumina DNA Prep with Enrichment (Illumina, San Diego, CA, USA), following manufacturer’s procedure, starting from 500 ng of DNA. Paired-end sequencing was performed using NextSeq 500 (Illumina, San Diego, CA, USA) with 101 bp of read length. Up to 10 libraries for WET were loaded on NextSeq High Output cartridge (300 cycles, Illumina).

### 2.14. Data Analysis

#### 2.14.1. FLIM Data Analysis

FLIM data, referenced with SimFCS 2, were analyzed using SimFCS 4, according to the protocol described by Ranjiit, in the section “Two-component analysis of fractional NADH distribution” [17]. From this analysis and as previously described [14], we obtained a mean NAD(P)H fractional distribution curve for treated GB-EXPs and one for control GB-EXPS. Comparing these two curves as reported in Morelli et al. [14], we calculated a percentage of drug response (%DR). Using %DR, samples were classified as: NR, non-responder: %DR < 5%; Resp, responder: DR ≥ 5%

#### 2.14.2. Next-Generation Sequencing Data Analysis

As a first step, RNA-Seq reads in FASTQ format were examined using the FASTQC program (http://www.bioinformatics.babraham.ac.uk/projects/fastqc/, accessed on 11 October 2023). Subsequently, alignment against the Hg19 reference genome was carried out using the STAR aligner (version 2.5.3a). The quantification of read counts on known human genes was accomplished utilizing feature Counts version 1.5.1. Differential expression analysis was performed using EdgeR 2.6.12 and Cuffdiff 2.2.0 tools. Differentially expressed genes (DEGs) were identified by intersecting the lists of significant DEGs (*p*-value < 0.004) obtained from both Cuffdiff 2 and edgeR.A discriminant stepwise analysis was used to find genes discriminating our two groups: Resp. and NR. JMP 10.0.0 (SAS Institute, Cary, NC, USA).

To compare the prognostic significance of our discriminant gene set in predicting survival in glioblastoma patients, we evaluated it in TCGA Database with SurvExpress platform. Heat-maps and PCA plots were generated using ClustVis version 2.0 (https://biit.cs.ut.ee/clustvis/, accessed on 4 December 2023) [18].

The initial analysis of exome data was conducted using the SeqMule pipeline [19]. For the detection of somatic single-nucleotide variants (SNVs) and indels within tumors, we employed a Panel of Normal (PoN) as recommended in the GATK best practices (https://console.cloud.google.com/storage/browser/gatk-best-practices/somatic-b37, accessed on 25 October 2023) along with the Mutect2 variant-calling algorithms [20]. Rare variants were acquired by filtering out somatic variants cataloged in the non-cancer database gnomAD v3 [21], with a minor allele frequency (MAF) threshold of ≥0.01. The frequency and characteristics of mutations were analyzed using the R package MAFtools version 2.18 [22].

Copy number analysis was conducted utilizing CNVkitt [23]. CNApp version 1.0 was employed with default cutoffs to summarize copy number variations [24]. Comparative data for the CNV classifier were obtained from The Cancer Genome Atlas Glioblastoma Multiforme (TCGA-GB, https://www.cancer.gov/tcga, accessed on 15 November 2023) dataset (hg19 Legacy Database) via the TCGAbiolinks version 2.30.0 [25] and randomForest 4.7-1.1 [26] R packages.

#### 2.14.3. Statistical Analysis

All presented summary data are expressed as means ± standard deviation (s.d.). Statistical analyses were conducted using R and GraphPad Prism software (GraphPad 7.0). Differences between two groups were assessed using Student’s two-tailed unpaired t-test or log-rank test, as specified in the figure legends. For *t*-tests, we assumed normality and equal distribution of variance among the different groups. No data points were excluded from the statistical analyses. Significance was defined as *p* < 0.05 (for all other experiments). Logistic regression analysis was performed with 5000 number of iteration and a learning rate of 0.005 with Statistic calculator DATAtab (https://datatab.net/statistics, accessed on 7 November 2023).

## 3. Results

### 3.1. Tumor Samples’ Characteristics

Tumors were collected from 18 patients, five of whom we had acquired both primary and recurrent tumor samples from. For the remaining patients, we only had recurrent samples, except for one patient wherein we only had the primary tumor. Additionally, for three patients, we obtained both core and peripheral portions from the same patient-derived tumor using neuronavigation-guided microsurgical techniques (Table 1). In total, we had 26 patient-derived surgical glioblastoma tissues. Patients included 13 men (72%) and 5 (28%) women in the age group of 52–79 years (mean age: 66 years). Information on cerebral localization and MGMT methylation status is reported in Table 1. All tumors were of wild type for IDH1/2 genes. The patient-derived tumor sample Gb14_pr showed the deletion of CDKN2A and CDKN2B and Gb15_pr reported the deletion of 1p.

### 3.2. FLIM Imaging of Patient-Derived Organoids

**Figure 1:**
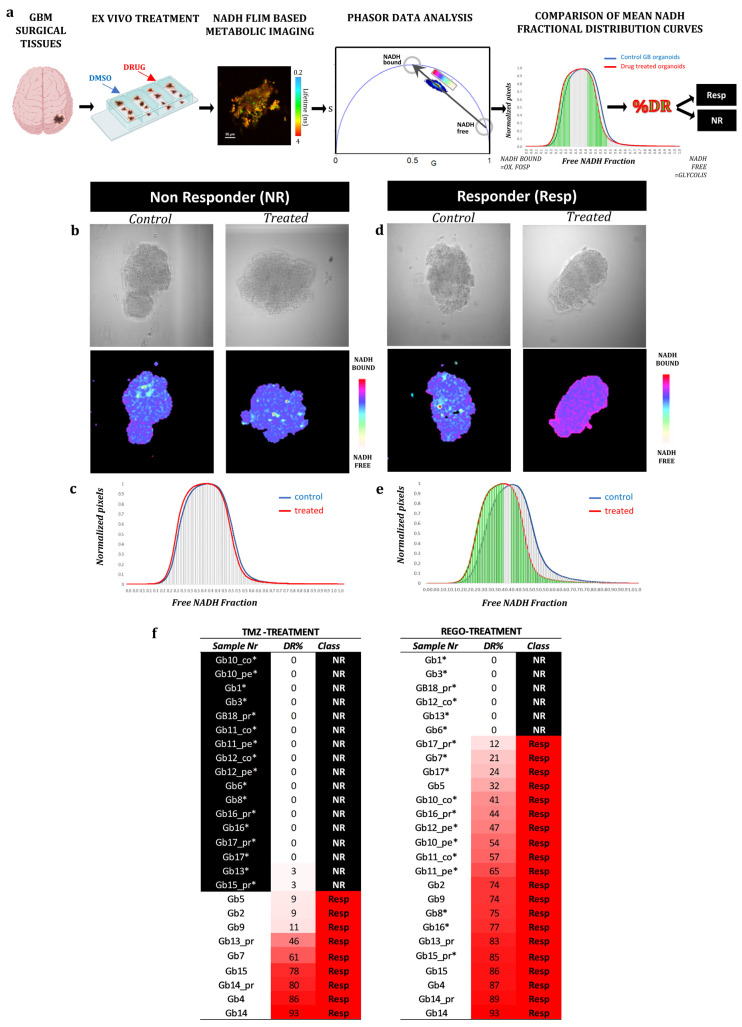
FLIM analysis of patient-derived GB-EXPs. (**a**) Workflow: Patient-derived GB-EXPs were obtained from surgical tissue, cultured in vitrogel, and treated for 72 h. FLIM analysis was performed on a range of 12 to 20 patient-derived GB-EXPs, including treated and control samples. Data were analyzed using the phasor approach, resulting in mean NAD(P)H fractional distribution curves for treated GB-EXPs (red) and control GB-EXPs (blue). The statistically significant leftward shift of the treated curve compared to the control curve is highlighted in green and indicates a more oxidative state in the treated GB-EXPs compared to the controls. The green area is indicative of the percentage of drug response (%DR). Tumors were stratified into non-responders (NRs, %DR ≤ 5) and responders (Resp, %DR > 5) using a cutoff of 5%DR. (**b**–**e**) Exemplary instances of one NR and one Resp tumor-derived GB-EXP post-treatment, featuring a brightfield image (**top**) and the corresponding phasor map (**bottom**). In the case of NRs, the NAD(P)H fractional mean distribution curves overlap between control (blue) and treated GB-EXPs (red), leading to a 0%DR (**c**). Conversely, in the case of Resp, the NAD(P)H fractional mean distribution curves exhibit a leftward shift of the red curves (treated GB-EXPs), resulting in a 74%DR (**e**). (**f**) Summary of %DR for all cases with both treatment modalities, TMZ (**left**) and REGO (**right**). The first column lists the sample identification number (Sample Nr), the second column displays the %DR, and the third column indicates the phenotype as NR or Resp. Cases are arranged from lower %DR at the top to higher %DR at the bottom, with color highlighting the %DR increase from white to red. NR sample cells are colored in black, with white characters; TMZ-NR samples are marked with an asterisk (*). Abbreviations: %DR, percentage of drug response; NR, non-responder; Resp, responder; cor, core portion of tumor; per, peripheral portion of the tumor; PR, primary tumor; REC, recurrent tumor.

From the 26 GB tissues collected, patient-derived organoids named GB-EXPs were obtained following the protocol described previously [14] and summarized in Figure 1a. GB-EXPs were derived from patient-derived tissue surgery, cultured in vitrogel, and treated with DMSO (controls), REGO, or TMZ for 72 h. A minimum of 12 to a maximum of 20 FLIM images were collected for each experimental condition and analyzed using the phasor approach to obtain mean NAD(P)H fractional distribution curves for controls and one for treated GB-EXPs. By comparing the two curves, we calculated a %DR (see Materials and Methods), represented as the green area under the treated curve. A cutoff of 5%DR was used, as previously described [14], to stratify tumors into non-responders (NRs) when DR ≤ 5% and responders (REGO) if DR > 5%. Considering the data obtained from the 2D and 3D cell line models (see Supplementary Results), we opted to use a REGO concentration of 50 µM for GB-EXP treatment. GB-EXPs were also treated with 600 µM of TMZ, as previously described [14]. All treatments lasted 72 h. Representative images of a GB-EXPs treated with 50 µM of REGO are shown in Figure 1b. The top row features a brightfield image, while the bottom row displays phasor-FLIM NAD(P)H lifetime maps. The coloration corresponds to the color bar defined on the side. Figure 1c shows the overlapping of the mean fractional NAD(P)H distribution curves of the control and Rego-treated patient-derived GB-EXPs, which is indicative of 0%DR, which indicates NR tumor. Conversely, in Figure 1d, we depict an instance of a GB-EXP, comprising a brightfield image (top row) and phasor-FLIM NAD(P)H lifetime maps (at the bottom). This demonstrates a statistically notable shift in the mean-treated curve (red) towards a higher prevalence of bound NAD(P)H molecular species in REGO-treated explants compared to the control counterparts, with 87% drug response (Figure 1e). Increased levels of bound-state NAD(P)H indicate oxidative metabolism, a characteristic of less proliferative cells, thereby signifying a responsive tumor [27,28]

The final annotation in Resp or NRs for each case and for both treatments (Rego and TMZ) leads us to stratify our tumor samples into 17 TMZ-NRs (65%) and 9 TMZ-Resp (35%), and 6 REGO-NRs (23%) and 20 REGO-Resp (77%) (Figure 1f). These data show a higher percentage of responders in Rego-treated tumors (72%) compared to TMZ-treated ones (39%). Particular attention must be paid to the fact that all 6 REGO-NRs were also TMZ-NRs; on the contrary, 6 out of 17 TMZ-NRs (35%) were REGO-Resp. Furthermore, in tumors treated with TMZ, both patient-derived samples from the peripheral (pe) and core (co) regions exhibited identical FLIM labels (NRs), indicating a uniform metabolic response to TMZ. In the case of REGO-treated tumors, within the three pairs of pe/co samples, one (Gb12) displayed a distinct response, with the core portion showing an NR phenotype while the peripheral region exhibited a responsive phenotype (Resp). This divergence in response likely corresponds to the more aggressive phenotype typically observed in the core portion compared to the peripheral region in GB tumors. Regarding the difference in drug response among primary and recurrence of the same patient, heterogeneity was observed in both TMZ- and REGO-treated cases, with two patient-derived primary/recurrence tumor pairs having a different response to TMZ (Gb13 and Gb15), and one to REGO (Gb13). It is noteworthy that the Gb13 primary tumor is more responsive both to TMZ and to REGO than its relative recurrence.

### 3.3. Mutational Genetic Background in Regorafenib Resp and NR GB Tumors

The mutational analysis conducted on 16 GB tumors before treatment, comprising 5 NR and 11 Resp cases, aimed to discern potential genetic distinctions between these two categories. We chose all mutations within the coding region known to affect protein function, along with all splicing mutations. Synonymous mutations with no predicted impact on splicing were excluded.

**Figure 2:**
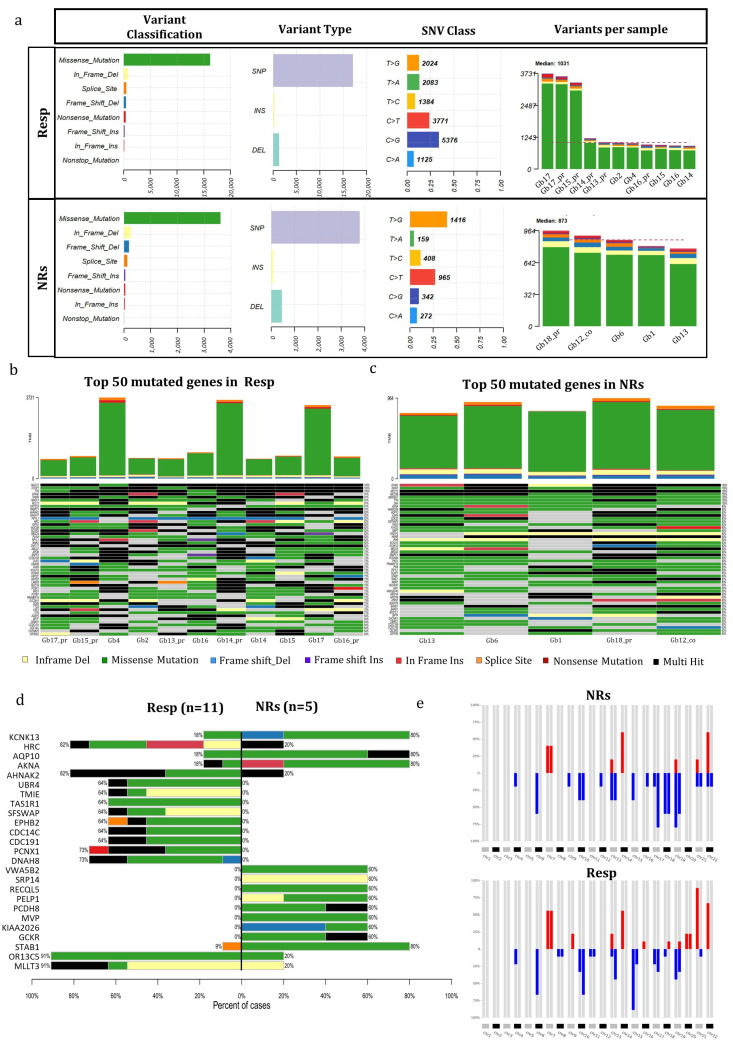
**Mutational analysis of GB patients**. (**a**) The mutation landscape of GB patients categorized as Resp (n = 11) and NRs (n = 5). This includes counts of each variant classification, variant type, single-nucleotide variant (SNV) classification, and the top 10 mutated genes. (**b**,**c**) Oncoplot illustrating the genes mutated in 100% (11/11) of Resp patients (**b**) and NR patients (**c**). (**d**) A co-bar plot indicating the genes significantly distinguishing between Resp and NR groups. Bars indicate the percentage of samples in which gene mutations were identified, with colors representing the types of mutation. (**e**) Heatmap displaying alterations in copy number of chromosome regions between Resp (n = 11) and NR (n = 5) groups (red indicates chromosome gains, and blue indicates losses). Abbreviations: Resp, responders; NRs, non-responders.

Figure 2a portrays the mutational landscape of both Resp and NR patients. The incidence of various types of mutations and the distribution of base substitutions exhibited remarkable parity between the two groups. However, a disparity emerged: C > G variations were notably more frequent in Resp (31%) compared to 10% in NR cases. Conversely, T > G variations prevailed in NRs (36%) as opposed to Resp (12%). The number of variants per sample was, on average, greater in the Resp group (1032) compared to the NR group (837) (Figure 2a). Among the Resp tumors, Gb17 exhibited the highest number of variants in both recurrence and primary tumors, while Gb15 showed the highest number only in primary one, with approximately 3200 variants. In contrast, Gb14 had the lowest number of variants, falling below the median. In NR samples, the number of variants per sample was more evenly distributed (Figure 2a), with Gb18_pr having the highest count and Gb13 having the lowest.

Figure 2b,c highlight the top 50 mutated genes that harbored mutations in every single case of Resp and NR patients. In the Resp group, three genes exhibited mutations in all samples, while the NR group showed mutations in seven genes across all cases. Notably, two genes, MUC12 and TTN, displayed mutations in 100% of both Resp and NR samples (see Figure 2b,c). Further, analysis of Table S1 reveals that both genes carry multiple mutations in most of the patients. In Figure 2d, a co-bar plot illustrates the genes that significantly distinguish the Resp and NR tumors. Remarkably, four genes—KCNK13, AQP10, AKNA, and STAB1—have been identified with mutations prevalent in the majority of NR samples (four out of five). In contrast, only two out of the eleven Resp samples exhibit mutations in the KCNK13, AQP10, and AKNA genes, while one out of eleven Resp samples carries the mutated STAB1 gene. Concerning the KCNK3 gene, all samples, except for Gb18_pr, share a common mutation (Val391Gly), with the exceptional case of Gb18_pr displaying a termination codon resulting from a frameshift starting at codon 88, as elucidated in Table S1. The gene AQP10 also exhibits the same mutation (ASN54Ser) across all samples. The remaining genes display a variety of molecular variations distributed among the different samples, as detailed in Table S1.

As depicted in Figure 2d, several genes exclusively exhibited mutations within the Resp group. Notably, two genes, PCNX1 and DNAH8, displayed mutations in 8 out of 11 Resp tumors, showcasing diverse mutations among samples (refer to Table S1). Remarkably, 91% of the Resp group (10 out of 11) exhibited mutations in the OR13C5 and MLLT3 genes, in contrast to the NR group, where only 20% (one out of five tumors) showed mutations. It is noteworthy that both genes harbored the same mutation in all samples; specifically, the OR13C5 gene exhibited a consistent multinucleotide variation affecting Threonine in codon 81 (Thr81), while an in-frame deletion consistently affected codon 190 (Ser190) in the MLLT3 gene.

### 3.4. Transcriptional Genetic Background in Regorafenib Resp and NR GB Tumors

WTA was performed on 13 GB-EXP samples that were REGO-treated, and on the 13 patient-corresponding GB-EXPs that were DMSO-treated (controls). Among these thirteen cases, nine were REGO-Resp and four NRs. A *p*-value < 0.001 and a log2-fold change greater than 0.05 or less than −0.05 were considered to identify differentially expressed genes (DEGs).

**Figure 3:**
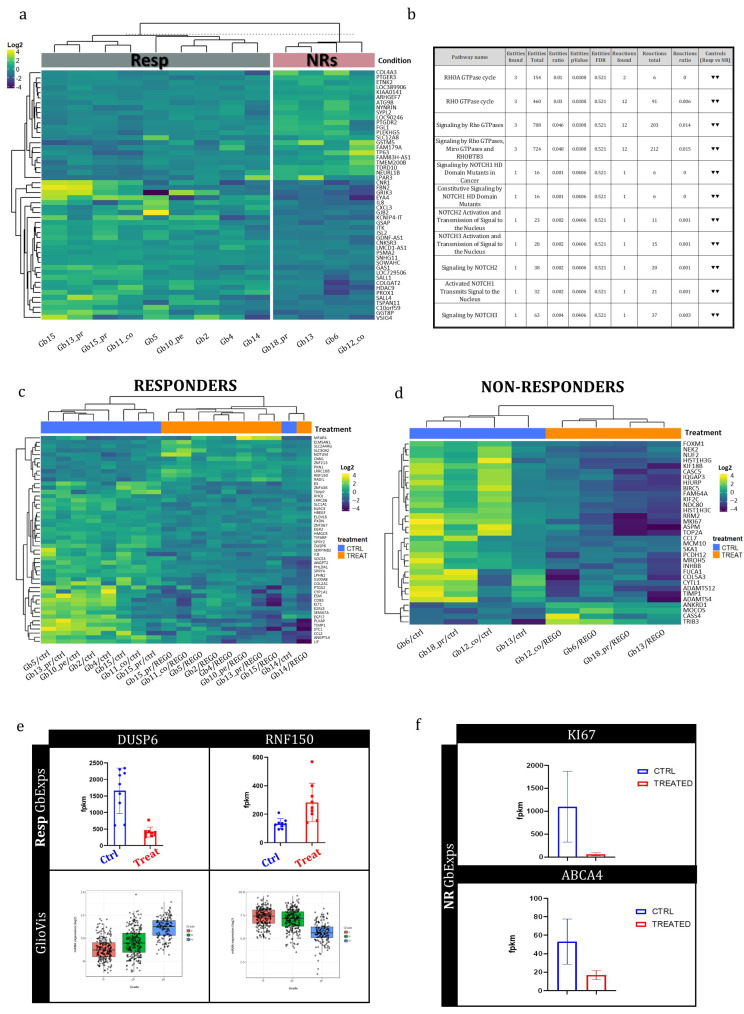
**Gene expression analysis of GB-EXPs**. (**a**) Heatmap of differentially expressed genes (DEGs) among Resp and NR samples in DMSO-treated GB-EXPs (controls). (**b**) Molecular pathways in which DEGsC are involved. (**c**) Heatmap of DEGs among controls and REGO-treated samples in responder GB-EXPs. (**d**) Heatmap of DEGs among controls and REGO-treated samples in NR GB-EXPs. (**e**) DUSP6 and RNF150 gene expression in GB-EXPs of Resp samples, and in GLIOVIS online database (TCGA samples and Agilent platform) according to tumor grade. (**f**) KI67 and ABCA4 gene expression in GB-EXPs of NR samples. Abbreviations: Resp, responders; NRs, non-responders; CTRL, controls; TREAT, treated GB-EXPs.

To understand the background expression pattern’s involvement in the response to REGO, we conducted differential expression analysis between Resp and NRs using the control samples. We identified 57 differentially expressed genes (Figure 3a). In particular, among these genes, we point out GRIK3, GSMT5, CXCL3, and SALL4 that are already involved in drug response mechanisms [29,30,31,32]. These four genes were significantly upregulated in the Resp tumors compared to the NR tumors except for GSTM5. Subsequently, we used the Reactome online tool to explore the molecular pathways associated with these DEGs. Our findings revealed that Rho GTPase and NOTCH signaling pathways were downregulated in Resp samples compared to NR samples (Figure 3b).

### 3.5. Early Whole-Transcriptome Changes Induced by Regorafenib Treatment in GB-EXPs

Whole-transcriptome analysis was performed to explore early gene expression changes occurring after REGO treatment in the Resp and NR samples. Even in this case, a *p*-value < 0.001 and a log2-fold change greater than 0.05 or less than −0.05 were considered to identify DEGs. Two classes of DEGs were obtained comparing control versus treated in Resp samples (DEGsA) and control versus treated in NR samples (DEGsB). Sixty and thirty-three DEGsA and DEGsB were found, respectively (Tables S2 and S3). In Figure 3c,d, heatmaps were run for Resp and NR samples. The results show that DEGsA and DEGsB perfectly classify the ctrls and the treated GB-EXP samples among the Resp and NR groups. Only one control sample in the Resp group (Gb14) was clustered among treated ones (Figure 3c). Upon analyzing the GLIOVIS database (http://gliovis.bioinfo.cnio.es/, accessed 4 December 2023), we observed that most of the downregulated DEGsA in treated GB-EXPs, compared to GB-EXP controls, as DUSP6, displayed an increased expression pattern as tumor grade increased (Figure 3e). This finding suggests that REGO treatment in Resp samples could potentially restore the expression of these genes to a pattern more similar to that of less aggressive tumor tissue. On the contrary, the only upregulated gene in treated GB-EXPs compared to controls, RNF150, showed an opposite expression pattern (Figure 3e) when compared to the downregulated DEGsA. This might indicate that Resp samples increased the expression of this gene, making it more similar to the expression pattern observed in less aggressive tumor tissue. Of the significantly dysregulated genes in the non-resp group (DEGsB), only 1 gene, KI67, demonstrated a significantly adjusted *p*-value of less than 0.05 (Figure 3f; Table S3). KI67, a well-known marker for proliferation [33], shows a statistically significant downregulation at 72 hr post REGO treatment compared to the non-treated sample. Among the genes typically involved in multidrug resistance [34], we found the drug transporter ABCA4 gene significantly downregulated after treatment in the NR group (Figure 3f), while no significant gene of the ABC family was found dysregulated in the Resp group.

To understand pathways in which DEGsA and DEGsB were involved, we queried the Reactome database (https://reactome.org/, accessed 15 December 2023). We found that DEGsA are primarily focused on MAP kinase (ERK/MAPK) signaling pathways inactivation/negative regulation in treated GB-EXPs when compared to control samples (Table S4), but also in interleukin-17 signaling, Toll-like receptor (TLR) cascades, and signaling by receptor tyrosine kinases (RTKs). On the other hand, DEGsB were centered around transcriptional regulation by E2F6, metabolic processes, membrane trafficking, and cell cycle regulation during the G2/M transition, underling the different response to REGO treatment in Resp and NR samples (Table S5).

## 4. Discussion

Glioblastoma (GB) is the most common malignant primary brain tumor in adults, with a survival period of post-standard treatment averaging 14.6 months [35,36]. Recurrence within 6–9 months after initial therapy is common, motivating exploration of multikinase inhibitors like Regorafenib (REGO) [11]. REGO targets angiogenic and oncogenic kinases [12]. Despite clinical use, the molecular mechanisms of REGO responsiveness in GB are poorly understood, underscoring the need for further research. Our study introduces a novel ex vivo precision medicine approach using 3D organoid models derived from patient GB tissue (GB-EXPs) [14]. These models enable the analysis of tissue responses to treatment. We focused on recurrent GB patients, comparing REGO and TMZ responses using NADH(P) fluorescence lifetime imaging [14,37]. We found higher REGO responders (77%) compared to TMZ (35%), with REGO non-responders (REGO-NRs) also being TMZ non-responders (100%). These data are consistent with results obtained from a study conducted on nude mice, which received subcutaneous inoculation with U87 cells and were treated with REGO (20 mg/kg/day) alone or with TMZ (10 mg/kg/day) [38]. The research found that 20 μM REGO induced a higher GB growth inhibition than 500 μM TMZ, and the cytotoxic impact of REGO was only minimally enhanced by TMZ. Additionally, the study confirmed these results in an orthotopic xenograft mouse model of human GB, showing that REGO significantly prolonged the survival of mice with orthotopic GB xenografts [38]. Our findings, when considered alongside this evidence, may suggest that REGO has greater efficacy in reducing tumor aggressiveness than TMZ. However, clinical validation is required to confirm our data on REGO response.

One of the most innovative approaches of this study was to analyze molecular changes of GB-EXPs after treatment through longitudinal in vitro tissue sampling, enabling a detailed examination of drug-induced molecular changes in a model closely resembling in vivo conditions. Initially, we conducted a comprehensive analysis of mutational profiles of REGO Resp and NR tumors. Within the NR group, we identified several genes with preferential mutation patterns, including KCNK3, AQP10, AKNA, and STAB1, potentially impacting protein functions. KCNK3, a member of the K+ channel protein family, has demonstrated significance in glioma growth, particularly given its extensive expression in the nervous system [39,40,41]. Aquaporins (AQPs), a family of water channel proteins, have been implicated in glioma pathogenesis, with overexpression associated with tumor severity and patient prognosis. The identification of AQP10 mutations suggests their potential relevance in glioma response to REGO treatment, warranting further investigation [42]. The AKNA gene, encoding a transcription factor with an enigmatic role in cancer, adds complexity to our understanding [43]. Additionally, the Scavenger Receptor Stabilin-1 (STAB1), identified as a receptor for the matricellular protein SPARC involved in glioblastoma cell migration, highlights its potential significance in cancer development and response to treatments like REGO, necessitating further exploration [44].

In the REGO-Resp group, notable molecular alterations were observed, particularly in the PCNX1, DNAH8, OR13C5, and MLLT3 genes. PCNX1, described as hypermutated in glioma, has somatic variants predictive of chemotherapy response in breast cancer [45]. DNAH8, associated with prostate and lung cancers, also correlates with increased tumor mutation burden (TMB), suggesting its potential as a predictive biomarker for immune checkpoint inhibitor therapy [46,47] OR13C5, a member of the odorant receptors (ORs) family, may have implications beyond odor perception, potentially influencing glioblastoma pathogenesis and serving as biomarkers or therapeutic targets [48]. MLLT3, frequently involved in gene fusions in leukemia and glioblastoma, presents a promising treatment target, given its oncogenic role and potential regulation of the JNK signaling pathway [49,50]. These diverse molecular alterations in the Resp group emphasize the complex genetic landscape influencing glioblastoma responses to REGO, suggesting potential avenues for targeted therapeutic interventions.

The comprehensive transcriptome analysis revealed differential expression of genes between Resp and NR GB-EXPs, highlighting GRIK3, GSMT5, and SALL4. GRIK3, encoding the glutamate receptor, is crucial in synaptic transmission and implicated in tumor-related pathways like MAPK/ERK and mTOR [30]. REGO’s modulation of similar channels in breast cancer suggests a potential therapeutic avenue for glioblastoma [51]. Notably, GRIK3 expression was significantly higher in Resp tumors, suggesting it as a potential REGO target. Decreased P2X7R gene expression in breast cancer cell lines post-REGO treatment aligns with our observations [51]. GSMT5, involved in drug detoxification, showed higher expression in REGO-resistant tumors, possibly indicating drug protection mechanisms [52]. SALL4, overexpressed in NR tumors, is associated with tumor progression and drug resistance, suggesting therapeutic potential [32]. Reactome analysis revealed downregulation of Rho GTPase and NOTCH pathways in Resp samples, consistent with higher REGO response [53,54]

The utilization of an essential in vitro model provided a valuable platform for studying gene expression changes induced by REGO drug treatment, with a focus on transcriptional alterations 72 h post-treatment. Comparative analyses between treated and non-treated tumors within the Resp and NR groups revealed a trend of general gene expression downregulation, consistent with the direction of most statistically significant deregulated genes. Notably, in the Resp group, DUSP6 and RFN50 genes were significantly modulated by REGO, resembling a genetic background typical of a lesser aggressive tumor, as reported in the GLIOVIS dataset (http://gliovis.bioinfo.cnio.es/, accessed on 4 December 2023). In the NR group, a striking observation was the significant 20-fold downregulation of MKI67, a known proliferation marker. Similar decreases in MKI67 expression have been reported in the literature [55]. Moreover, it should be noted that temporarily pausing cell division (dormancy) could be advantageous for cancer cell survival [56]. Although it seems unlikely for cancer cells to enter dormancy on their own, harsh conditions, like limited growth factors and immune system attacks, might force them into this state, emphasizing the complexity of drug responses and the need for a nuanced exploration of molecular pathways involved in resistance [56].

In cancer cells, multidrug resistance (MDR) significantly undermines therapeutic effectiveness, often attributed to overexpression of ATP-binding cassette (ABC) transporter proteins [34]. Our analyses identified ABCA4 as the only gene modulated by REGO treatment, with significantly reduced expression in the NR group, potentially depriving cells of drug uptake.

To elucidate pathways associated with DEGsA and DEGsB, we conducted a comprehensive analysis using the Reactome database. DEGsA primarily engage in attenuating/negatively regulating MAP kinase (ERK/MAPK) signaling pathways, interleukin-17 signaling, Toll-like receptor (TLR) cascades, and signaling by receptor tyrosine kinases (RTKs), potentially contributing to drug response mechanisms to REGO [57,58]. DEGsB focus on transcriptional regulation by E2F6, metabolic processes, membrane trafficking, and cell cycle regulation during the G2/M transition [59].

## 5. Conclusions

Our findings suggest that using our innovative approach, which allows for the study of longitudinal molecular alterations, it may be possible to identify patients who are more likely to respond to a treatment based on their tumor’s molecular profile. This could lead to the development of new therapies that target these pathways more effectively and to more personalized treatment decisions that could improve outcomes for patients with glioblastoma.

## Figures and Tables

**Table 1:** Tumor samples’ description. All tumors were recurrent, except those indicated with the suffix _pr, which denotes primary tumors. For recurrent tumors with both peripheral and core portions available (Y = yes), they were marked with the suffixes _pe and _co, respectively. In the column “Tumor Sample Portion Available”, the type of sample (primary or recurrence) is specified, along with the portion of the recurrence (peripheral and core).

Patient Nr	Sex	Age at Intervention	Tumor Nr	Tumor Sample Portion Available				Localization
				Pr	Rec	Co	Pe	
**Pt.01**	**m**	57	Gb1		Y			right frontal
**Pt.02**	**m**	73	Gb2		Y			left temporal
**Pt.03**	**m**	52	Gb3		Y			left temporal
**Pt.04**	**m**	73	Gb4		Y			left temporal–parietal
**Pt.05**	**m**	73	Gb5		Y			left occipital
**Pt.06**	**f**	57	Gb6		Y			right temporal
**Pt.07**	**f**	62	Gb7		Y			right parietal
**Pt.08**	**m**	61	Gb8		Y			left frontoparietal–temporal
**Pt.09**	**m**	56	Gb9		Y			
**Pt.10**	**m**	70	Gb10_co		Y	Y		left temporal–parietal
			Gb10_pe		Y		Y	
**Pt.11**	**m**	70	Gb11_co		Y	Y		left fusiform and parahippocampal gyrus
			Gb11_pe		Y		Y	
**Pt.12**	**f**	69	Gb12_co		Y	Y		left frontoparietal
			Gb12_pe		Y		Y	
**Pt.13**	**m**	55	Gb13_pr	Y				right frontal
		56	Gb13		Y			right frontal
**Pt.14**	**m**	72	Gb14_pr	Y				left frontal and insular
		73	Gb14		Y			left temporal
**Pt.15**	**f**	74	Gb15_pr	Y				left temporal
		75	Gb15		Y			left frontotemporal
**Pt.16**	**m**	78	Gb16_pr	Y				left temporal
		79	*Gb16*		Y			left temporal
**Pt.17**	**f**	56	Gb17_pr	Y				left hemisphere multifocal
		58	Gb17		Y			left temporal
**Pt.18**	**m**	76	Gb18_pr	Y				left temporal

## Data Availability

All data generated or analyzed during this study are included in this published article and its supplementary information files. The datasets used and/or analyzed during the current study are available from the corresponding author on reasonable request.

## Supplementary Materials

The following supporting information can be downloaded at: https://www.mdpi.com/article/10.3390/cells13060487/s1, Figure S1: Viability and proliferation effects of Regorafenib on 2D and 3D cell line models, T98G, U87 and U118; Figure S2: FLIM analysis on 3D-T98G cell line using different REGO concentrations; Supplementary Table S1: Exome matrix in glioblastoma bulk samples; Supplementary Table S2: Differentially expressed genes among Regorafenib-treated group and controls in responder GB-EXP samples; Supplementary Table S3: Differentially expressed genes among Regorafenib-treated group and controls in non-responder GB-EXPs samples; Supplementary Table S4: Reactome pathways of differentially expressed genes among Regorafenib-treated group and controls in responder GB-EXP samples; Supplementary Table S5: Reactome pathways of differentially expressed genes among Regorafenib-treated group and controls in non-responder GB-EXP samples; Supplementary Results: Sensitivity to Regorafenib in 2D and 3D commercial cell lines.
