# Supplementary material for: Exploring Regorafenib Responsiveness and Uncovering Molecular Mechanisms in Recurrent Glioblastoma Tumors through Longitudinal In Vitro Sampling
Source: Cells. 2024 Mar 11;13(6):487. doi: 10.3390/cells13060487 (PMC10968984; doi:10.3390/cells13060487)
Supplement: Supplementary file 1 [file cells-13-00487-s001.zip › Supplementary Table S2.pdf]

Supplementary Table S2. Differentially Expressed Genes among Regorafenib treated and controls in Responders GB-EXPs samples.

Gene	BaseMean	log2FoldChange	lfcSE	stat	pvalue	padj	Regulation
DUSP6	1030.602564	-2.035445528	0.295084956	-6.89782887	5.28E-12	6.31E-08	down
SPRY4	1025.179366	-1.478026206	0.29532205	-5.004794626	5.59E-07	0.003342994	down
TIPARP	1485.264829	-1.08733372	0.22733749	-4.782905456	1.73E-06	0.006885835	down
PLVAP	374.1973502	-3.343906939	0.711454307	-4.700100774	2.60E-06	0.007772391	down
F3	1659.06811	-1.83031619	0.39667464	-4.614149743	3.95E-06	0.009438253	down
STC1	1068.707797	-3.107891567	0.701243146	-4.431974252	9.34E-06	0.01860636	down
NLRC3	307.7242286	-1.342031415	0.309403705	-4.337476881	1.44E-05	0.024617009	down
ESM1	354.0899544	-2.848268339	0.661832088	-4.30361173	1.68E-05	0.025112987	down
SEMA7A	576.6733932	-2.007412305	0.475993701	-4.21730855	2.47E-05	0.032843872	down
SLC30A2	27.04120244	2.607940852	0.625101515	4.172027728	3.02E-05	NA	up
SERPINB2	9.381732009	-6.584501841	1.606558547	-4.098513469	4.16E-05	NA	down
HMGCR	632.7932346	-0.990838182	0.2434905	-4.069309405	4.72E-05	0.049908631	down
PHLDA1	4306.481294	-1.310959456	0.322990581	-4.058816372	4.93E-05	0.049908631	down
RNF150	208.085889	1.08354345	0.267198793	4.0551959	5.01E-05	0.049908631	up
PKN1	2717.81574	0.752678426	0.192713147	3.905693198	9.40E-05	0.086410295	up
LRRC36	32.31731943	-2.292724878	0.590940929	-3.879786904	0.000104548	NA	down
ANGPTL4	2115.89789	-1.901486534	0.490842613	-3.873923095	0.000107097	0.088282202	down
ELOVL6	228.9735214	-0.916711606	0.237183323	-3.864991827	0.000111093	0.088282202	down
EGR2	311.5887651	-0.954647682	0.248228459	-3.845843005	0.000120139	0.088282202	down
LRRC16B	240.334553	1.338450465	0.349002553	3.835073566	0.000125527	0.088282202	up
SRSF7	704.6769414	-0.466772455	0.122238602	-3.818535622	0.000134246	0.08916931	no
IL8	1156.482835	-1.733954275	0.456649529	-3.797122662	0.000146385	0.090037756	down
SPRY2	877.5602359	-0.981474459	0.25896062	-3.790052938	0.000150615	0.090037756	down
F2RL3	34.21939241	-2.800868893	0.739860116	-3.78567358	0.000153293	NA	down
MFAP4	578.0008982	2.552818153	0.679762667	3.755455065	0.000173027	0.098509927	up
PXDNL	29.34698962	-1.315393254	0.350541893	-3.75245664	0.00017511	NA	down
CNN1	43.6354944	1.546722655	0.41364811	3.739223314	0.00018459	NA	up
HBEGF	404.0392227	-0.697221523	0.186586813	-3.736713825	0.000186441	0.101322159	down
EGFL7	734.0982389	-1.978845734	0.532420474	-3.716697292	0.000201844	0.102200272	down
ANGPT2	590.9991496	-1.558032639	0.419662433	-3.71258544	0.000205153	0.102200272	down
PTGS2	545.6326945	-2.216381476	0.6000597	-3.693601616	0.0002211	0.103370079	down
LPHN2	838.7193936	-0.923627119	0.250346864	-3.689389606	0.000224793	0.103370079	down
LIF	1401.853848	-1.979614009	0.538176781	-3.67837127	0.000234728	0.103941085	down
SLC1A1	151.5108207	-1.18666296	0.32530571	-3.647839326	0.000264455	0.112922253	down
RADIL	286.2457902	1.216666558	0.337625843	3.603594286	0.000313847	0.12939147	up
ELMSAN1	3222.752259	1.248463265	0.348761063	3.579709431	0.000343976	0.137086092	up
TXNIP	2910.060855	-1.600517255	0.450399513	-3.553550143	0.000380069	0.1456154	down
FLT1	1343.418986	-2.100758216	0.592273788	-3.546937682	0.000389737	0.1456154	down
TIMP1	3587.147659	-2.166666219	0.612718344	-3.536153669	0.000405998	0.147094471	down
COL2A1	37.75100552	-3.216793486	0.916664829	-3.509236293	0.000449395	NA	down
NOTUM	48.80689011	2.645944532	0.759444475	3.484052646	0.000493882	NA	up
SLC2A4RG	925.5863233	0.938749279	0.270002792	3.476813236	0.000507411	0.174931366	up
CCL2	1686.438647	-2.215222925	0.637593636	-3.474349176	0.000512094	0.174931366	down
S100A8	27.32945055	-2.701345784	0.777607717	-3.473918435	0.000512917	NA	down
CYP1A1	160.1947662	-2.619788196	0.755940558	-3.465600791	0.000529048	0.175702781	down
RHOJ	613.1190672	-1.017387222	0.295772668	-3.439760777	0.000582229	0.188138508	down
SOCS3	872.8901328	-1.374404393	0.402590318	-3.413903242	0.000640393	0.201487905	down
ZNF367	122.5972361	-0.771004796	0.226724943	-3.400617444	0.000672338	0.206114841	down
ZNF213	405.4122695	0.540739015	0.160080888	3.377911146	0.000730387	0.211001929	up
ZNF436	1343.904538	-1.302383716	0.385785273	-3.375929068	0.000735669	0.211001929	down
CD93	1157.460239	-2.240097254	0.663957084	-3.373858505	0.000741225	0.211001929	down
EXOSC6	562.2297023	0.570603493	0.169937102	3.35773345	0.000785844	0.214932099	up
TGM2	1303.208826	-2.024361916	0.603984698	-3.351677486	0.000803235	0.214932099	down
HKDC1	131.7487337	2.348666598	0.701155167	3.349710176	0.000808962	0.214932099	up
LOC100287015	40.88701543	-0.720844041	0.217012353	-3.321672847	0.000894795	NA	down
TFPI2	220.7361122	-1.820005468	0.549455193	-3.312381961	0.000925052	0.23464676	down
PARP6	1245.405129	0.580339577	0.175488533	3.306994296	0.000943028	0.23464676	up
ZNF205	256.8796368	0.579945649	0.175843627	3.298076011	0.000973498	0.23464676	up
DLL4	245.6145378	-1.757148405	0.533601945	-3.292994756	0.000991263	0.23464676	down
C22orf23	606.4132535	1.310984578	0.398216788	3.292137897	0.000994288	0.23464676	up
