# Supplementary material for: Exploring Regorafenib Responsiveness and Uncovering Molecular Mechanisms in Recurrent Glioblastoma Tumors through Longitudinal In Vitro Sampling
Source: Cells. 2024 Mar 11;13(6):487. doi: 10.3390/cells13060487 (PMC10968984; doi:10.3390/cells13060487)
Supplement: Supplementary file 1 [file cells-13-00487-s001.zip › Supplementary Table S3.pdf]

Supplementary Table S3. Differentially Expressed Genes among Regorafenib treated and controls in Non Responders GB-EXPs samples.

Gene	baseMean	log2FoldChange	lfcSE	stat	pvalue	padj	Regulation
MKI67	577.6869245	-4.22796691	0.695351	-6.08033	1.20E-09	2.56E-05	down
TOP2A	641.8904594	-3.619006931	0.809008	-4.47339	7.70E-06	0.061511616	down
CASC5	250.7372044	-3.234736383	0.745442	-4.33935	1.43E-05	0.061511616	down
BIRC5	123.3057132	-3.568481032	0.822436	-4.33892	1.43E-05	0.061511616	down
HJURP	117.0095012	-3.688975215	0.853645	-4.32144	1.55E-05	0.061511616	down
KIF18B	123.3869476	-3.683588732	0.857212	-4.29718	1.73E-05	0.061511616	down
CCL7	12.15598713	-6.991931175	1.72184	-4.06073	4.89E-05	0.143992438	down
NDC80	53.1565635	-3.478099681	0.869127	-4.00183	6.29E-05	0.143992438	down
MOCOS	94.3891931	2.324618091	0.584021	3.980366	6.88E-05	0.143992438	up
NUF2	54.58216328	-2.664170168	0.671068	-3.97004	7.19E-05	0.143992438	down
NEK2	40.14613204	-3.298374363	0.832445	-3.96227	7.42E-05	0.143992438	down
MROH5	64.70807534	-3.652838069	0.935541	-3.90452	9.44E-05	0.167858458	down
HIST1H3G	38.29670344	-5.485509234	1.42629	-3.846	0.00012	0.183637514	down
RRM2	171.1417248	-3.529479473	0.917916	-3.8451	0.000121	0.183637514	down
IQGAP3	297.7613924	-2.610891753	0.707047	-3.69267	0.000222	0.296686936	down
HIST1H3C	49.49353902	-3.322823526	0.900006	-3.692	0.000222	0.296686936	down
INHBB	201.66417	-2.40361943	0.654143	-3.67446	0.000238	0.299135967	down
FAM64A	33.76801197	-3.362778599	0.91907	-3.65889	0.000253	0.300239476	down
KIF2C	134.1409627	-2.582654225	0.712757	-3.62347	0.000291	0.318428171	down
FUCA1	1522.267364	-2.885756273	0.797921	-3.6166	0.000299	0.318428171	down
FOXM1	173.5181152	-1.941039219	0.543807	-3.56935	0.000358	0.355617559	down
ANKRD1	12.49935044	3.031102022	0.850727	3.562956	0.000367	0.355617559	up
TRIB3	1188.796628	2.561106621	0.726689	3.524349	0.000425	0.39379262	up
ADAMTS4	1943.209037	-2.945731879	0.841239	-3.50166	0.000462	0.401536278	down
TIMP1	3530.528891	-2.774024281	0.793257	-3.497	0.000471	0.401536278	down
ADAMTS12	263.5787597	-2.991143026	0.858622	-3.48365	0.000495	0.405872723	down
CASS4	56.37722042	3.162495154	0.910874	3.471935	0.000517	0.408306131	up
PCDH12	552.3489498	-2.295062687	0.664607	-3.45326	0.000554	0.416925999	down
COL5A3	2706.82181	-2.994533799	0.86872	-3.44706	0.000567	0.416925999	down
CYTL1	45.45230986	-3.956604456	1.161088	-3.40767	0.000655	0.464903135	down
SKA1	44.05699295	-2.227608081	0.655308	-3.39933	0.000676	0.464903135	down
ASPM	509.4167206	-3.196657133	0.963774	-3.31681	0.000911	0.607052102	down
MCM10	39.33890565	-2.313319127	0.70197	-3.29547	0.000983	0.63438137	down
