# Supplementary material for: Exploring Regorafenib Responsiveness and Uncovering Molecular Mechanisms in Recurrent Glioblastoma Tumors through Longitudinal In Vitro Sampling
Source: Cells. 2024 Mar 11;13(6):487. doi: 10.3390/cells13060487 (PMC10968984; doi:10.3390/cells13060487)
Supplement: Supplementary file 1 [file cells-13-00487-s001.zip › Supplementary Table S4.pdf]

Supplementary Table S4. Reactome pathways of Differentially Expressed Genes among Regorafenib treated and controls in Responders GB-EXPs samples.

Pathway name	Entities found	Entities Total	Entities ratio	Entities pValue	Entities FDR	Reactions found	Reactions total	Reactions ratio	Responders (treated vs ctrl)
ERKs are inactivated	1	15	0.001	1.00E-05	1.45E-04	*1*	*2*	*0*	▲▲
ERK/MAPK targets	1	26	0.002	1.00E-05	1.45E-04	*1*	*7*	*0*	▲▲
RAF-independent MAPK1/3 activation	1	28	0.002	1.00E-05	1.45E-04	*1*	*12*	*0.001*	▲▲
MAPK targets/ Nuclear events mediated by MAP kinases	1	35	0.002	1.00E-05	1.45E-04	*1*	*16*	*0.001*	▲▲
Negative regulation of MAPK pathway	1	49	0.003	1.00E-05	1.45E-04	*1*	*17*	*0.001*	▲▲
MAP kinase activation	1	69	0.005	1.00E-05	1.45E-04	*1*	*32*	*0.002*	▲▲
Interleukin-17 signaling	1	78	0.005	1.00E-05	1.45E-04	*1*	*35*	*0.002*	▲▲
RAF/MAP kinase cascade	2	322	0.021	1.00E-05	1.45E-04	*2*	*75*	*0.005*	▲▲
MAPK1/MAPK3 signaling	2	329	0.022	1.00E-05	1.45E-04	*2*	*82*	*0.006*	▲▲
Oncogenic MAPK signaling	1	93	0.006	1.00E-05	1.45E-04	*1*	*46*	*0.003*	▲▲
TRAF6 mediated induction of NFkB and MAP kinases upon TLR7/8 or 9 activation	1	116	0.008	1.00E-05	1.45E-04	*1*	*60*	*0.004*	▲▲
MAPK family signaling cascades	2	380	0.025	1.00E-05	1.45E-04	*2*	*122*	*0.009*	▲▲
MyD88 cascade initiated on plasma membrane	1	109	0.007	1.00E-05	1.45E-04	*1*	*70*	*0.005*	▲▲
TRIF(TICAM1)-mediated TLR4 signaling	1	121	0.008	1.00E-05	1.45E-04	*1*	*70*	*0.005*	▲▲
Toll Like Receptor 10 (TLR10) Cascade	1	109	0.007	1.00E-05	1.45E-04	*1*	*71*	*0.005*	▲▲
Toll Like Receptor 5 (TLR5) Cascade	1	109	0.007	1.00E-05	1.45E-04	*1*	*71*	*0.005*	▲▲
MyD88-independent TLR4 cascade	1	121	0.008	1.00E-05	1.45E-04	*1*	*72*	*0.005*	▲▲
Toll Like Receptor 3 (TLR3) Cascade	1	116	0.008	1.00E-05	1.45E-04	*1*	*73*	*0.005*	▲▲
MyD88 dependent cascade initiated on endosome	1	117	0.008	1.00E-05	1.45E-04	*1*	*75*	*0.005*	▲▲
Toll Like Receptor 7/8 (TLR7/8) Cascade	1	118	0.008	1.00E-05	1.45E-04	*1*	*79*	*0.006*	▲▲
Toll Like Receptor 9 (TLR9) Cascade	1	121	0.008	1.00E-05	1.45E-04	*1*	*80*	*0.006*	▲▲
Diseases of signal transduction by growth factor receptors and second messengers	3	498	0.033	1.80E-02	2.50E-01	*25*	*478*	*0.033*	▲
Signaling by Receptor Tyrosine Kinases	9	623	0.041	2.30E-02	3.07E-01	*100*	*746*	*0.052*	▲▲
Nuclear Events (kinase and transcription factor activation)	5	80	0.005	4.10E-02	4.86E-01	*14*	*48*	*0.003*	▲▲
Signaling by NTRK1 (TRKA)	5	143	0.009	4.10E-02	4.86E-01	*14*	*102*	*0.007*	▲▲
Signaling by NTRKs	5	166	0.011	4.10E-02	4.86E-01	*14*	*164*	*0.011*	▲▲
