# Supplementary material for: Exploring Regorafenib Responsiveness and Uncovering Molecular Mechanisms in Recurrent Glioblastoma Tumors through Longitudinal In Vitro Sampling
Source: Cells. 2024 Mar 11;13(6):487. doi: 10.3390/cells13060487 (PMC10968984; doi:10.3390/cells13060487)
Supplement: Supplementary file 1 [file cells-13-00487-s001.zip › Supplementary Table S5.pdf]

Supplementary Table S5. Reactome pathways of Differentially Expressed Genes among Regorafenib treated and controls in Non Responders GB-EXPs samples.

Pathway name	Entities found	Entities Total	Entities ratio	Entities pValue	Entities FDR	Reactions found	Reactions total	Reactions ratio	Non Responders (treated vs ctrl)
Transcriptional Regulation by E2F6	2	46	0.003	1.45E-04	7.22E-03	*2*	*33*	*0.002*	▲▲
Interconversion of nucleotide di- and triphosphates	1	80	0.005	1.45E-04	7.22E-03	*2*	*33*	*0.002*	▲▲
Metabolism of nucleotides	1	256	0.017	1.45E-04	7.22E-03	*2*	*142*	*0.01*	▲▲
Metabolism	7	3.647	0.239	1.45E-04	7.22E-03	*6*	*2.268*	*0.159*	▼
Kinesins	2	68	0.004	2.90E-02	5.16E-01	*3*	*14*	*0.001*	▲
COPI-dependent Golgi-to-ER retrograde traffic	2	107	0.007	2.90E-02	5.16E-01	*2*	*11*	*0.001*	▲
Molybdenum cofactor biosynthesis	1	27	0.002	2.90E-02	5.16E-01	*1*	*6*	*0*	▼
Golgi-to-ER retrograde transport	2	148	0.01	2.90E-02	5.16E-01	*2*	*18*	*0.001*	▲
Intra-Golgi and retrograde Golgi-to-ER traffic	2	219	0.014	2.90E-02	5.16E-01	*2*	*48*	*0.003*	▲
Membrane Trafficking	2	668	0.044	2.90E-02	5.16E-01	*2*	*219*	*0.015*	▲
Vesicle-mediated transport	2	828	0.054	2.90E-02	5.16E-01	*2*	*252*	*0.018*	▲
Metabolism of water-soluble vitamins and cofactors	1	258	0.017	2.90E-02	5.16E-01	*1*	*143*	*0.01*	▼
Metabolism of vitamins and cofactors	1	382	0.025	2.90E-02	5.16E-01	*1*	*205*	*0.014*	▼
Polo-like kinase mediated events	1	23	0.002	4.35E-02	6.01E-01	*12*	*15*	*0.001*	▲
G2/M Transition	2	212	0.014	4.35E-02	6.01E-01	*26*	*78*	*0.005*	▲
Mitotic G2-G2/M phases	2	214	0.014	4.35E-02	6.01E-01	*26*	*80*	*0.006*	▲
Cyclin A/B1/B2 associated events during G2/M transition	1	32	0.002	4.35E-02	6.01E-01	*2*	*25*	*0.002*	▲
