# Supplementary figures and images for: Exploring Regorafenib Responsiveness and Uncovering Molecular Mechanisms in Recurrent Glioblastoma Tumors through Longitudinal In Vitro Sampling
Source: Cells. 2024 Mar 11;13(6):487. doi: 10.3390/cells13060487 (PMC10968984; doi:10.3390/cells13060487)

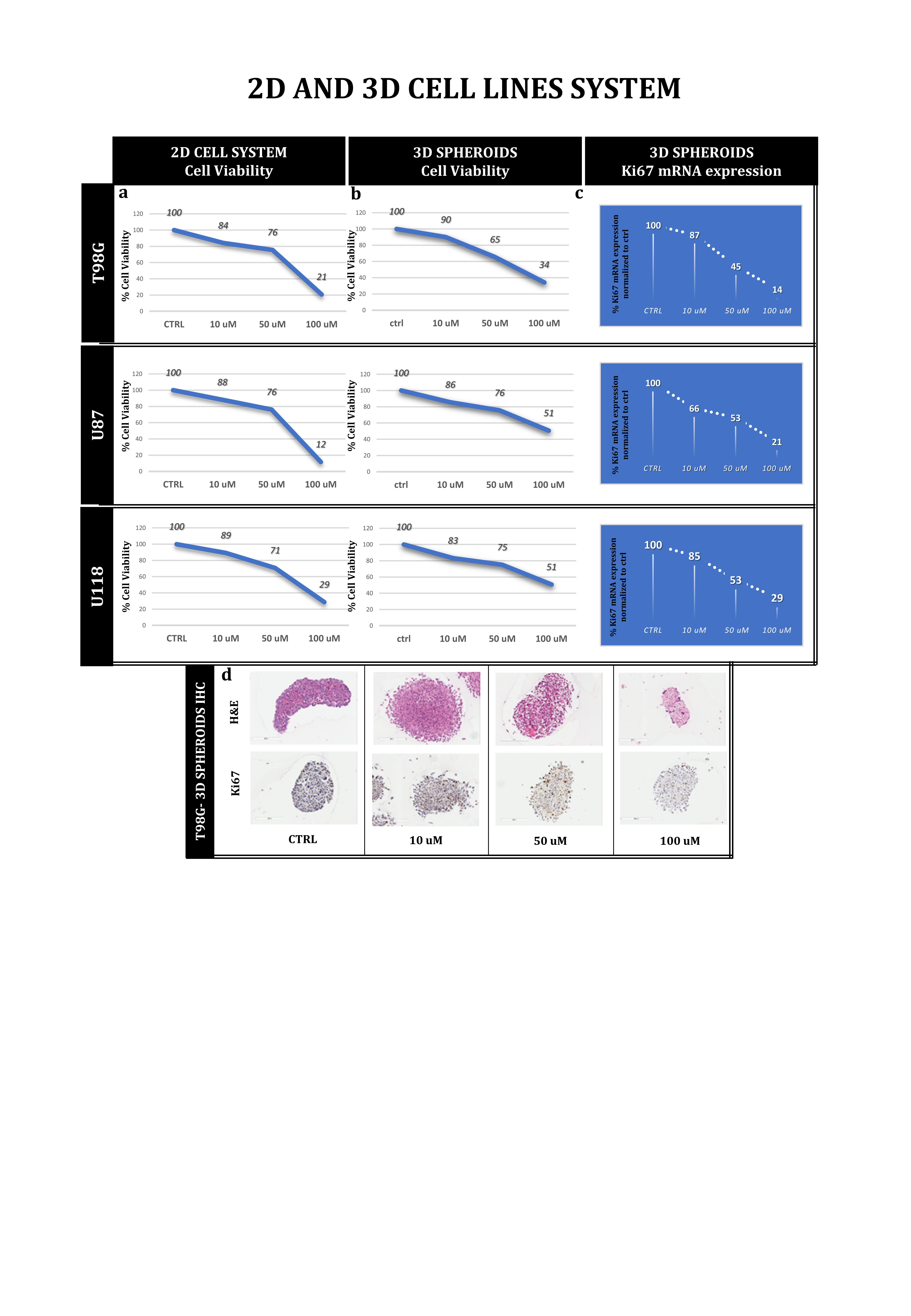

Supplement: Supplementary file 1 [file cells-13-00487-s001.zip › Figure S1.png]

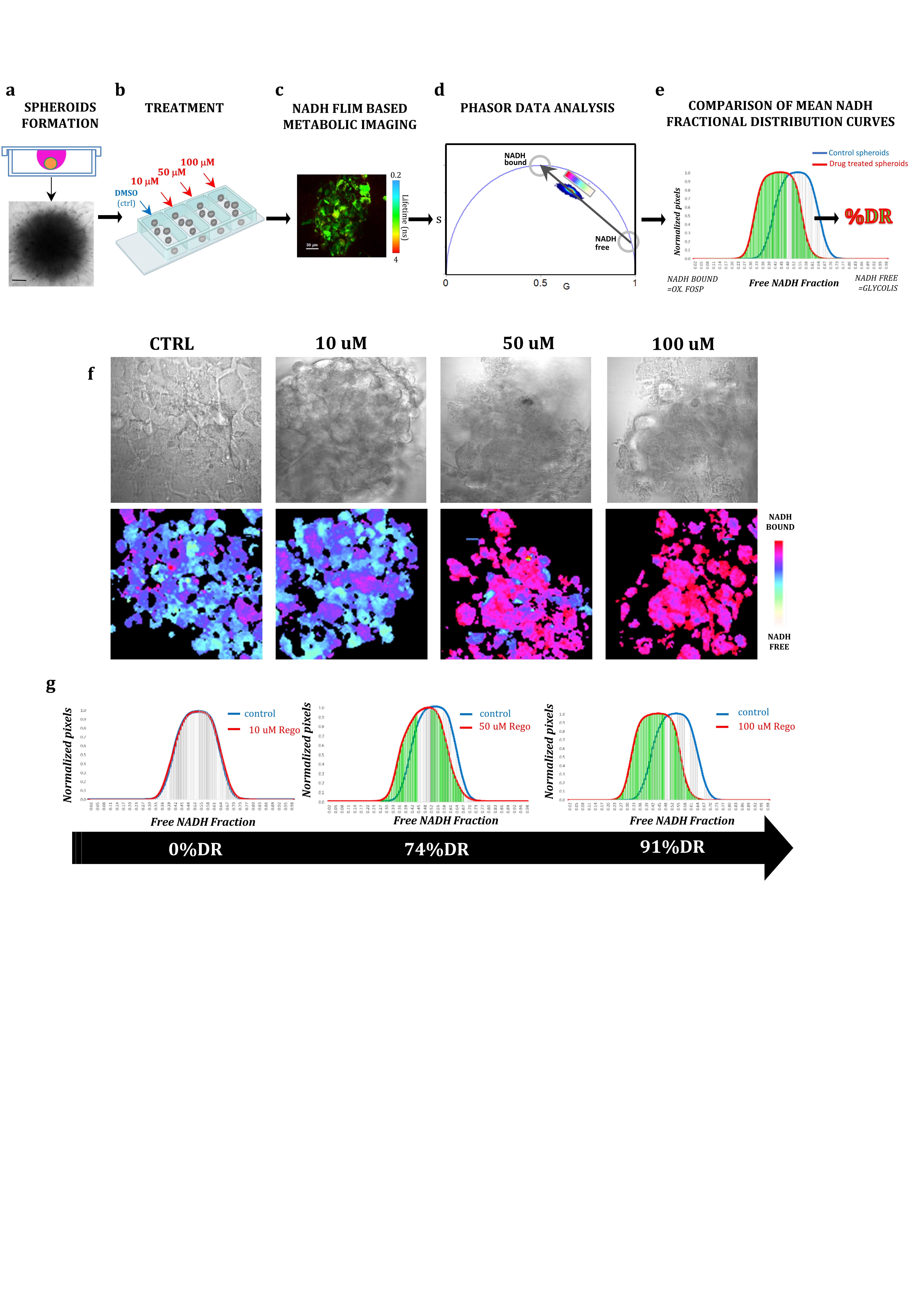

Supplement: Supplementary file 1 [file cells-13-00487-s001.zip › Figure S2.png]
